# French “real life” experience of clofarabine in children with refractory or relapsed acute lymphoblastic leukaemia

**DOI:** 10.1186/2162-3619-1-39

**Published:** 2012-12-10

**Authors:** Pascale Trioche, Brigitte Nelken, Gérard Michel, Isabelle Pellier, Arnaud Petit, Yves Bertrand, Pierre Rohrlich, Claudine Schmitt, Nicolas Sirvent, Patrick Boutard, Geneviève Margueritte, Brigitte Pautard, Stéphane Ducassou, Dominique Plantaz, Alain Robert, Caroline Thomas, Kristell Desseaux, Sylvie Chevret, André Baruchel

**Affiliations:** 1 Department of Pediatric, APHP, Hôpital Antoine Béclère, Service de Pédiatrie, 157 rue de la porte de Trivaux, 92141 Clamart Cedex, France; 2 CHU, Lille, France; 3 Hôpital La Timone, APHM, Marseille, France; 4 CHU, Angers, France; 5 APHP, Hôpital Armand Trousseau, Paris, France; 6 CHU, Lyon, France; 7 CHU, Besançon, France; 8 CHU, Nancy, France; 9 CHU, Nice, France; 10 CHU, Caen, France; 11 CHU, Montpellier, France; 12 CHU, Amiens, France; 13 CHU, Bordeaux, France; 14 CHU, Grenoble, France; 15 CHU, Toulouse, France; 16 CHU, Nantes, France; 17 APHP, Hôpital Saint-Louis, Département de Biostatistique et Informatique Médicale, Paris et Université Paris Diderot, Paris, France; 18 APHP, Hôpital Robert Debré, Hématologie et Immunologie Pédiatrique, Paris, et Université Paris Diderot, Paris, France

**Keywords:** Acute lymphoblastic leukaemia, Childhood leukaemia, Refractory, Relapsed, Leukaemia, Clofarabine

## Abstract

**Background:**

Clofarabine alone or in combination with cyclophosphamide and etoposide has shown a good efficacy and a tolerable toxicity profile in previous studies of children with relapsed or refractory leukaemia. This report describes a retrospective study of 38 French patients who received clofarabine as a monotherapy or in combination for relapsed or refractory acute lymphoblastic leukaemia (ALL) outside of clinical trials after marketing authorization.

**Methods:**

We retrospectively analysed data for 38 patients, up to 21 years old, attending 17 French centres. Thirty patients received clofarabine alone or in combination for a bone marrow relapse of acute lymphoblastic leukaemia (ALL) or refractory disease and eight patients for a high level of minimal residual disease (MRD). Survival and response durations were estimated by the Kaplan-Meier method.

**Results:**

For the 30 patients who received clofarabine for a bone marrow relapse of ALL (number of relapse, 1-3; median, 1), the overall remission rate (ORR) was 37%: eight complete remission (CR) and three complete remission without platelet recovery (CRp). Ten of the 11 responding patients subsequently underwent haematopoietic stem cell transplantation (HSCT).

Only four of the eight patients who received clofarabine while in remission for a high level of MRD, showed a moderate improvement of MRD. Seven of these eight patients received HSCT and six of them were alive at the end of the study. One other patient was alive without receiving HSCT.

However, clofarabine treatment was associated with a high risk of infection and hepatotoxicity. Febrile neutropenia grade ≥ 3 was reported in 79% of patients and documented infections grade ≥ 3 occurred in nine patients (24%). Hepatotoxicity grade 3 was reported in nine patients (24%). We observed four deaths related to treatment.

**Conclusion:**

In our experience, the efficacy of clofarabine is poorer than previously reported. Its toxicity is high and can be life threatening. Prospective studies on clofarabine used during earlier phases of the disease may help to define how best this new drug can be exploited for childhood and adolescent ALL.

## Background

Acute lymphoblastic leukaemia (ALL) is the most common form of cancer in children, responsible for approximately 30 percent of all childhood malignancies. Despite substantial improvements in the survival of children with ALL over the last thirty to forty years, 20 to 25% of children relapse [1,2]. Many children with relapsed ALL achieve a second remission, but the overall final outcome remains unsatisfactory, with long-term overall survival rates ranging from 15 to 50% [3]. Treatment for relapsed ALL primarily involves many of the same chemotherapy agents used initially, and also haematopoietic stem cell transplantation (HSCT) [4-6]. Because of the very poor prognosis of these patients treated with conventional therapy, innovative treatment strategies based on the use of novel anti-leukaemic agents are needed.

Antimetabolites are some of the most effective drugs against haematological malignancies. One of these new antimetabolic drugs is clofarabine (2-chloro-9(2’-deoxy-2’-fluoro-β-D-arabinofuranosyl)adenine), a novel second-generation purine nucleoside analogue synthesized with the aim to overcome the limitations (neurotoxicity), but to retain the useful properties, of fludarabine and cladribine [7-11]. Its antitumour activity is due to three mechanisms: inhibition of DNA polymerase α, inhibition of ribonucleotide reductase and disruption of mitochondrial membrane integrity causing the release of proapoptotic factors leading to programmed cell death even in non-dividing lymphocytes.

Phase I and II studies with clofarabine as a single-agent have been conducted in paediatric and adult patients with multiple relapse or refractory leukaemia [12-16]. These studies have shown the safety and the efficacy of clofarabine with an overall remission rate of 20% in paediatric patients. The most frequently observed grade ≥ 3 adverse events were febrile neutropenia, hypokalaemia, elevated aspartate or alanine transaminase levels, hyperbilirubinaemia and neutropenia. Systemic inflammatory response-like or cytokine release-like events, skin rash and hand-foot syndrome were also observed.

In view of these results, clofarabine was approved by both the Food and Drug Administration in the United States and the European Medicinal Evaluation Agency for the treatment of ALL in paediatric patients (≤ 21 years old) who have relapsed or are refractory after receiving at least two prior regimens and for who no other treatment option can be expected to result in a durable response [15].

Clofarabine inhibits the repair of DNA damage. Using it in combination with alkylating agents such as cyclophosphamide would therefore be expected to result in enhanced cytotoxic effects. Indeed, a synergistic effect of this type was demonstrated *in vitro*[17] and was subsequently confirmed in clinical trials [18]. Phase I and II studies with a combination of clofarabine, cyclophosphamide and etoposide were performed by Hijiya et al. and Locatelli et al. [19,20]. The results were very encouraging with overall remission rates (ORR) of 55% and 56%, respectively, for patients with relapsed or refractory ALL. The most common adverse events were the same as those found for clofarabine used as a single agent. In some cases, however, the use of clofarabine in combination was associated with severe and potentially life-threatening hepatotoxicity [19].

This paper reports a retrospective study of 38 French patients who received clofarabine as monotherapy or as part of combination therapy. The patients had relapsed or refractory ALL (30 cases) or were in remission but with a high MRD (8 cases).

## Methods

### Study group

Data were obtained for 38 patients from 17 paediatric hemato-oncology French centres. This study was approved by the Leukaemia Committee of the SFCE (*Société Française de lutte contre les Cancers et les leucémies de l’Enfant et de l’adolescent*). Anonymised data were collected using a standardised Case Report Form.

All patients who received at least one course of clofarabine for relapsed or refractory ALL (30 cases) or for a high MRD despite remission (8 cases), between January 1, 2006 and January 1, 2010, and who were less than 21 years old at the time of treatment were included. No other eligibility criteria were required. For all the patients, demographic data, leukaemia characteristics, previous treatments, mode of administration of clofarabine, adverse events and outcome after clofarabine therapy were collected.

The cut-off date for this analysis was September 1, 2010.

### Treatment

Clofarabine was administered alone to nine patients at a dose of 52 mg/m^2^ daily for five days. Twenty-nine patients received clofarabine in combination. Three different combinations were used. The two most widely used combinations were 40 mg/m^2^/day clofarabine, 440 mg/m^2^/day cyclophosphamide and 100 mg/m^2^/day etoposide, as reported in the study by Hijiya et al. (21 patients) [19] and 40 mg/m^2^/day clofarabine, 400 mg/m^2^/day cyclophosphamide and 150 mg/m^2^/day etoposide as reported in the study by Locatelli et al. (5 patients) [20]. For these two treatment combinations, all drugs were administered for five consecutive days. The three remaining patients received clofarabine in association with another chemotherapy. Most patients (27 of 38) received only one course of clofarabine; seven of the 38 patients received two courses of clofarabine and four received three courses.

### Response and toxicity criteria

Complete remission (CR) was defined as no detectable leukaemia cells in peripheral blood, M1 bone marrow (≤ 5% blasts) and recovery of peripheral counts (platelets ≥ 100 x 10^9^/l and an absolute granulocyte count (ANC) ≥ 1.0 x 10^9^/l). CR in the absence of platelet recovery (CRp) was defined as patients who met all the criteria for a CR except for the recovery of platelet counts (platelets < 100 x 10^9^/l). Partial remission (PR) was defined as complete disappearance of circulating blasts and either a M2 bone marrow (> 5% and ≤ 25% blasts) and appearance of normal progenitor cells or a M1 bone marrow that did not qualify for CR or CRp. The overall remission rate (ORR) was defined as the number of patients who achieved CR or CRp divided by the number of patients treated.

MRD was analysed by real-time quantitative-polymerase chain reaction (RT-qPCR) analysis of “leukaemia-specific” junctional regions of rearranged immunoglobulin (Ig) genes and T-cell receptor (TCR) genes, according to the ESG-MRD-ALL guidelines 2007 [21].

Adverse events (AEs) were evaluated using National Cancer Institute’s Common Terminology Criteria for Adverse Events (NCI CTCAE v4.0).

### Statistical analysis

Quantitative variables are reported as medians and ranges, and qualitative variables as percentages. Fisher’s test was used for comparison of response rates. Factors predictive of remission were assessed through univariable logistic regression models and the odds ratio (OR) with ninety-five percent confidence intervals (95%CI) are reported.

The durations of survival and of response were estimated by the Kaplan-Meier method; patients who did not experience the event concerned were censored at the date of last follow-up. Survival curves were compared using the log-rank test.

All statistical analyses were performed on R software ( http://www.R-project.org). All statistical tests were two-sided with type I error of 0.05.

## Results

### Patients and treatment

The patient characteristics at initial diagnosis are shown in Table 1. Median age at diagnosis was 4 years (range, 0-16); three patients were diagnosed when infants. There were 22 boys and 16 girls. The initial diagnosis was B lineage ALL for 33 patients, T cell ALL for two patients and a biphenotypic ALL for the remaining three. The median white blood cell count was 7.45 x 10^9^/l (range, 0.9-675). Only three patients with B lineage ALL had high-risk cytogenetic features (one t(4; 11); two with hypodiploidy defined as less than 45 chromosomes). Two patients had central nervous system (CNS) involvement at diagnosis. The initial treatment protocol was EORTC 58951 for 17 patients and FRALLE 2000 for 17 patients. Three patients were included in the Interfant 2006 protocol. The last patient (biclonal T cell and myeloid leukaemia) was treated in the acute myeloid leukaemia protocol (ELAM02). All patients were in CR after primary therapy.

**Table 1:** Patient characteristics (N=38)

**Patient characteristics**	**Number (%)**
Gender: male/female	22/16 (58%/42%)
Median age at diagnosis, years (range)	4 (0-16)
Median age at clofarabine treatment onset, years (range)	7 (0-18)
WBC count at diagnosis x10^9^/l (range)	7.45 (0.9-675)
Immunophenotype	
B lineage	33 (87%)
T lineage	2 (5%)
Biphenotypic	3 (8%)
CNS involvement at diagnosis	2
Adverse cytogenetics	
t(4;11)	1
Hypodiploid karyotype	2
First line protocol	
FRALLE 2000	17
EORTC 58951	17
ELAM 02	1
Interfant 2006	3
Refractory to initial treatment	0
Number of earlier therapies, mean (range)	2.5 (1-4)
Previous hematopoietic stem-cell transplantation	10

**Figure 1:**
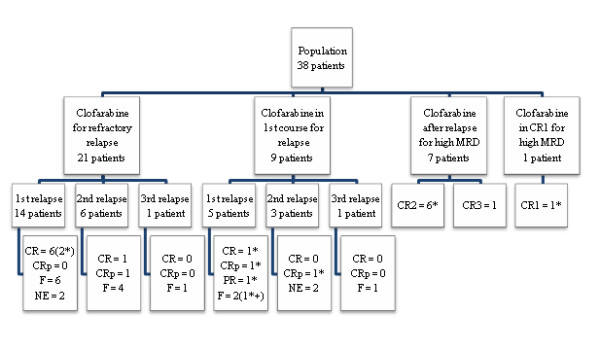
**Study flowchart.** CR, complete remission; CRp, complete remission without platelet recovery; PR, partial remission; F, failure; NE, not evaluable; MRD, minimal residual disease; * indicates patients alive at the end of the study; + indicates patients in palliative treatment.

Thirty patients received clofarabine for a bone marrow relapse of ALL and eight for a high MRD (seven patients after relapse treatment and one patient for a high MRD in CR1) (Figure 1). Of the 30 patients treated for a relapse of ALL, nine patients (30%) received clofarabine as the first treatment for relapse and twenty-one patients (70%) had a refractory relapse with at least one previous treatment for the relapse before clofarabine.

**Figure 2:**
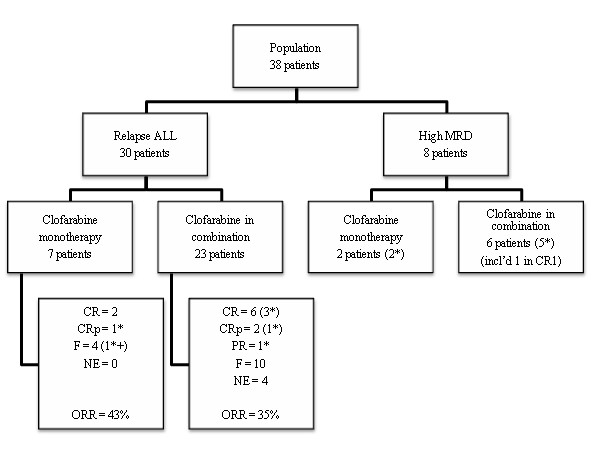
**Treatment flowchart.** CR, complete remission; CRp, complete remission without platelet recovery; PR, partial remission; F, failure; NE, not evaluable; MRD, minimal residual disease; * indicates patients alive at the end of the study; + indicates patients in palliative treatment.

Nine patients were treated with clofarabine as monotherapy: two for high MRD and seven for a relapse of disease. The 29 other patients received clofarabine in combination, six for high MRD and 23 for relapse (Figure 2).

The median interval between initial diagnosis and clofarabine administration was 1.8 year for the 30 patients treated for a relapse of ALL and 2.7 years for the eight patients treated for a high MRD.

Overall, these 38 patients were heavily pre-treated with a mean of 2.5 prior therapies before clofarabine (range, 1-4). Ten patients had received a prior HSCT (five in CR1 and five in CR2).

### Response and outcome

To describe the response to treatment, the population was separated in two groups: one group of 30 patients treated for a bone marrow relapse of ALL and a second group of eight patients treated for a high MRD.

In the first group of 30 patients, eight achieved CR and three CRp, giving an overall remission rate (ORR) of 37%. Twenty-one received clofarabine for a refractory relapse with at least one previous treatment for the relapse before clofarabine and nine as first treatment for relapse. Note that these 30 patients had been heavily pre-treated with a mean of 2.5 prior therapies, as for the whole population. Eight patients achieved remission after the first cycle of clofarabine, two patients after two cycles and one after three cycles. The initial diagnosis was B lineage ALL for 26 patients, T cell ALL for two patients and biphenotypic ALL for the last two. Ten of the 26 children (38%) with B ALL and one of the two children with T ALL achieved CR or CRp. The two cases of biphenotypic leukaemia failed to respond. Remission was obtained for eight of the 21 patients with refractory relapse (7 CR and 1 CRp; ORR 38%). For the nine patients who received clofarabine as the first treatment for relapse (five for a first relapse, three for a second relapse and one for a third relapse), the ORR was 33% (1 CR and 2 CRp). Seven patients received clofarabine as a monotherapy and 23 received clofarabine in combination. The ORR did not differ between the combination regimens (35%) and the use as a single agent (43%) (p=1.00) (Figure 2). In this first group of 30 patients, three had adverse cytogenetic abnormalities and failed to respond to clofarabine treatment. The probability of responding to treatment was not influenced by age at diagnosis (p=0.94) or WBC count at diagnosis (p=0.63) but only by the time interval between the diagnosis and the first relapse (p=0.02) (Additional file 1: Table S1). Two of the eight patients (25%) who had previously received HSCT responded to clofarabine treatment, and nine (41%) of the 22 patients who had not been previously transplanted responded.

Among these 30 patients, five received clofarabine as the first treatment for first bone marrow relapse. These five patients presented an ORR of 40% (1CR and 1CRp). They had particularly severe ALL (three of them with a prior HSCT in CR1, early relapses for all five patients, and one patient with severe hypodiploidy). However, one of these five patients who was in a PR after clofarabine treatment reached CR under further administration of conventional chemotherapy.

Ten of the 11 responding patients subsequently received HSCT. The median interval between clofarabine treatment and HSCT was 3.5 months (range, 3.1-4.3). Five of the ten transplanted patients were alive at the end of the study (range, 4.5-22 months).

For the 19 non-responding patients, two were alive at the end of the study (one was in PR after clofarabine and the other was being given palliative treatment).

The second group of eight patients treated for high MRD, had, as expected, a higher survival rate than the first group. However, the MRD levels did not decrease significantly after clofarabine therapy (Table 2). Only one patient had an improvement of more than one log in MRD (≥10^-2^ before clofarabine and <10^-3^ after clofarabine). This patient was treated in first remission with a combination including clofarabine. Seven of these eight patients subsequently received HSCT (only one patient received chemotherapy after clofarabine treatment and before HSCT) and six of them were alive at the end of the study. The eighth patient is alive without having received HSCT.

**Table 2:** Impact of clofarabine on patients with a high MRD (N=8)

**MRD evolution**	**Number (%)**
Improvement of one logarithm	1 (12.5%)
Improvement of less than one logarithm	3 (37.5%)
Stable	3 (37.5%)
Non evaluable	1 (12.5%)

**Figure 3:**
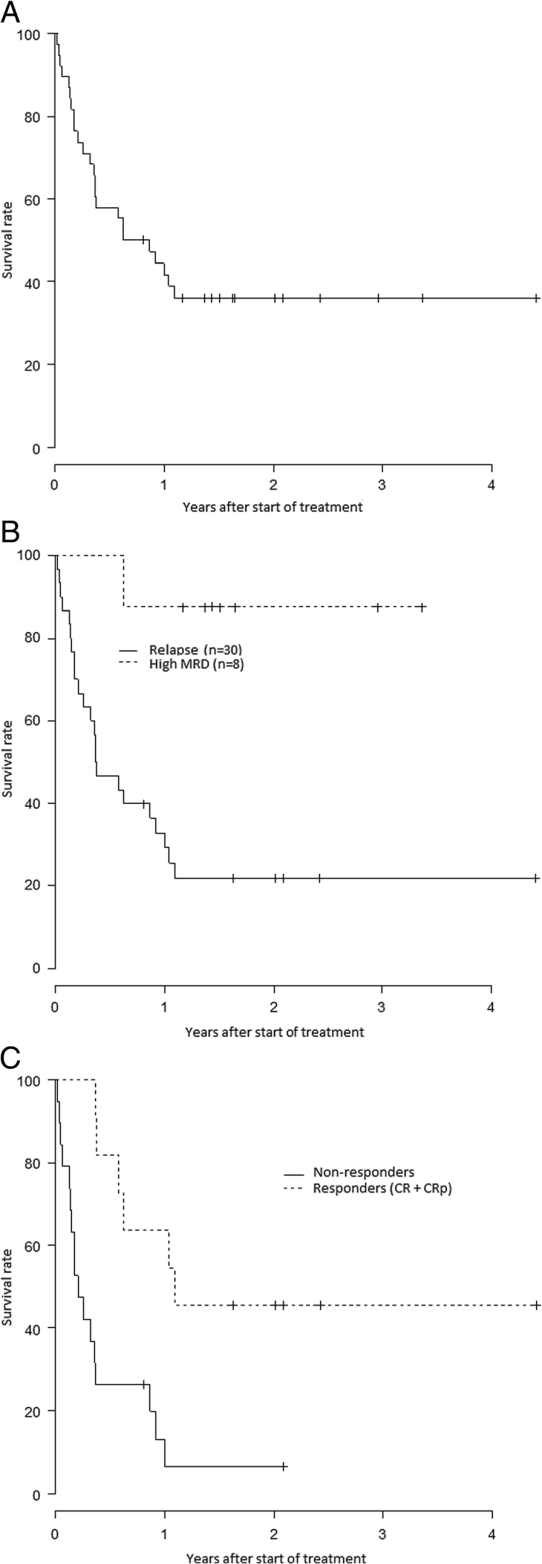
**Overall survival (Kaplan-Meier).** Figure 3**A** Overall survival for the 38 patients from the beginning of clofarabine treatment Figure 3**B** Overall survival for the 30 patients with relapse of ALL versus the 8 patients treated for a high MRD Figure 3**C** Overall survival for the 30 patients with relapse of ALL from start of treatment by response to treatment.

The Kaplan-Meier curves for overall survival (OS) are shown in Figure 3. The probability of survival one year after the beginning of clofarabine treatment for the entire cohort of patients was 38.1% (95%CI: 13.7%-100%). Patients treated for high MRD had a significantly higher probability of survival than those treated for relapsed ALL (with 1 year OS estimated at 87.5%, 95%CI: 67.3%-100% versus 29.1%, 95%CI: 16.5%-51.4%) (p=0.003). Among the 30 patients treated for relapse, the 1 year survival rates for patients who did or did not achieve CR or CRp were 63.6% (95%CI: 40.7%-99.5%) and 6.6% (95%CI: 1%-42.1%) (p=0.0015), respectively.

### Toxicity

Treatment-related toxicities are presented in Table 3. Only side effects of grade ≥ 3 are reported. Febrile neutropenia grade ≥ 3 was reported in 79% of patients. Documented infections grade ≥ 3 occurred in nine patients (24%); six of these nine patients developed septicaemia (16%) and three pneumonia (8%). Infectious complications were more frequent in patients treated with clofarabine in combination than alone (27.5% versus 11%). The median time to neutrophil recovery (defined as an absolute granulocyte count > 0.5 x 10^9^/l), known for only 10 of the 11 patients who achieved CR or CRp, was 22.5 days (range, 15.75-31). Nine patients (24%) had hepatotoxicity grade 3; eight patients had elevated transaminases (one with CR2 previous HSCT), one patient had elevated transaminases and hyperbilirubinaemia (without previous HSCT). No case of veno-occlusive disease (VOD) was noted. Hepatotoxicity did not differ significantly between patients treated with clofarabine alone or in combination. Acute renal failure was diagnosed in four patients: one grade 3 and three grade 4 (2 had concomitant multi-organ failure). The deaths of four patients (10.5%) were potentially associated with treatment-related complications. All four patients developed fatal multi-organ failure. One patient presented with refractory first relapse despite two treatments and was in a bad general state before starting clofarabine treatment. Another patient received clofarabine for a refractory first relapse; he had a severe hypodiploidy with 25 chromosomes and had previously suffered severe toxicity during first line therapy after methotrexate treatment (MTHFR mutation). The third patient had received clofarabine for a second bone marrow relapse and had undergone previous HSCT in CR2. The fourth patient presented with a second combined relapse (medullary and CNS) with paraplegia and blastic meningitis before the beginning of clofarabine treatment. All these four patients had received clofarabine in combination and two of them received concomitant intrathecal chemotherapy.

**Table 3:** Treatment-related toxicity: NCI CTCAE v4.0 (N=38)

**Adverse events (grade ≥3)**	**Number of patients (%)**
Febrile neutropenia	30 (79%)
Hepatic dysfunction	9 (24%)
Diarrhea	8 (21%)
Vomiting	7 (18%)
Hypokalemia	7 (18%)
Bacterial sepsis	6 (16%)
Mucositis	4 (10.5%)
Headache	4 (10.5%)
Multi-organ failure	4 (10.5%)
Lung infection	3 (8%)

## Discussion

This multicentre French retrospective study of 38 patients treated with clofarabine for ALL shows results below expectations. In the group of 30 patients who received clofarabine for an ALL in relapse, the ORR obtained was only 37%. This value is lower than those of 55% reported by Hijiya et al. [19] and 56% by Locatelli et al. [20] for patients treated with a combination of clofarabine, cyclophosphamide and etoposide. However, these phase I and/or II studies were conducted with patients who met the eligibility criteria. We obtained better results than studies with clofarabine used alone, where remission rates are about 20% [12,14-16]. Two additional studies have been recently published. The study by O’Connor, similar to ours, presents the UK experience of clofarabine in the treatment of relapsed and refractory paediatric ALL, and they obtain an ORR of 52% [22]. The second is the phase II study of Hijiya [23], and he reports an ORR of 44%. However, in our study, most of patients had advanced disease when clofarabine was administered. They were heavily pre-treated, with an average of 2.5 treatments before clofarabine, and 70% of them were refractory to their most recent prior treatment. These factors may have contributed to the low ORR we observed. Patients in the studies by O’Connor and Hijiya had received a mean of only 2 or 2.1 previous treatments, respectively. Hijiya reports that 60% of the patients were refractory to their most recent treatment [23], but no information was provided about refractory patients in the O’Connor report [22]. Only one study published recently, reports lower results than ours for a population of refractory or relapsed ALL [24]. Nevertheless, this result must be interpreted with caution due to the small number of patients included (9).

Surprisingly, the ORR was similar for refractory patients (ORR 38%) and those treated with clofarabine as the first line for a relapse (33%). Also, the ORR was not better for the five patients treated with clofarabine for a first relapse and who had only received one prior treatment (ORR 40%). The results obtained for these five patients differ from those reported by O’Connor [22]. Effectively, in the same conditions, O’Connor reported an ORR of 86% (7 patients, 6 CR). Obviously, all these results must be interpreted with caution due to the small numbers of patients involved.

Ten of the 11 patients in remission after clofarabine therapy subsequently underwent HSCT (90%) and five of these ten were alive (50%) at the end of data analysis with a median follow-up post HSCT of 13 months (range; 4.5-22). These data are similar to those reported by other studies with transplantation rates for patients in remission after clofarabine of 50% for Locatelli et al. [20], 54% for Hijiya et al. [19], 63% for Hijiya et al. [23] and 83% for O’Connor et al. [22] The survival rates after HSCT for responding patients were 57% (4/7 patients), in the study by Locatelli with a median follow-up of 8 months after HSCT [20], and 60% (6/10 patients) in the study by O’Connor, with a median follow-up of 13 months post HSCT [22]. Finally, the proportion of patients in remission after clofarabine was lower in our cohort than in other studies, but the post-clofarabine transplantation rate and survival rate were similar to other published values.

Eight patients in complete remission but with a high MRD level were treated by clofarabine. But clofarabine treatment only moderately improved (one log or less) the MRD levels in four of these eight patients. Most of these patients were transplanted after clofarabine treatment and the survival rate in this sub-group was high (87.5% one year after clofarabine). It is not possible to draw informative conclusions because of the small number of patients in this subset. Inaba et al. [24] reported two cases treated for a high MRD, and both showed a significant improvement of their MRD.

Adverse events associated with clofarabine treatment are frequent. In our study, clofarabine was generally well tolerated. However, we report four deaths (10.5%) that could potentially be attributed to the treatment. These four patients were all in poor condition and developed multi-organ failure after clofarabine treatment. Hijiya et al. [19] report a death rate of 8% (2 patients) after clofarabine treatment, but no deaths were reported by Locatelli et al. [20] or O’Connor et al. [22]. In their phase II study, Hijiya et al. [23] reported a substantial drug-related death rate of 24% (6/25 patients) due notably to severe hepatotoxicity with VOD.

For other adverse events, the incidences we observed were similar to published data. The incidence of febrile neutropenia in our study (79%) is comparable with those reported previously: 60-65% [19,22,23]. We had fewer documented infections (grade ≥3) than in other studies, with only nine cases (24%) including six of sepsis. Infection rates reported previously are in a range of 30-72% [19,20,22] depending on supportive therapy and infection prophylaxis. Thus, infectious complications are frequent with clofarabine treatment. To decrease their incidence, prophylactic treatments against bacterial and fungal infections with an active monitoring of patients are required.

Hepatic toxicity is frequent when clofarabine is used as single agent or in combination [12,14,18]. Some severe and life-threatening cases of hepatic toxicity were described by Hijiya et al. [19,23] (3 VOD, 1 hyperbilirubinaemia). In our study, grade 3 hepatotoxicity occurred in nine patients (24%; 1 hyperbilirubinaemia and elevated transaminase and 8 elevated transaminases), but there were no cases of VOD. The hepatotoxicity rate in our study was not higher among patients with previous HSCT than among those without HSCT.

## Conclusion

This French “real life” experience of clofarabine treatment for refractory or relapsed ALL reveals slightly lower response rates than previously reports. Our remission rates were somewhat lower than those presented in other studies. However, we observed a transplantation rate and survival rate similar to those of other published studies. The other common idea to use clofarabine for MRD reduction in the pre-transplant phase is not substantiated here. Nevertheless, the numbers of patients studied are small, and their disease severe, such that additional studies are required to determine the best use of clofarabine. It could be informative to use clofarabine at an earlier stage of the disease, to assess if it is more effective earlier during disease progression. Indeed, the next International Study on Relapsed ALL will randomize the use of combinations including clofarabine for patients at high risk.

Clofarabine is associated with a high rate of infection and hepatotoxicity. Deaths related to treatment were observed. These risks must be taken into consideration before clofarabine treatment is administered. Finally, the cost of this new drug makes it essential to conduct comparative and prospective evaluations of its risk-benefit ratio.

## Competing interests

AB is a member of the Board of the International Clofarabine Registry, funded by Genzyme. All other authors declare that they have no competing interest.

## Authors’ contributions

AB initiated the study concept and design. PT was responsible for the study execution and coordination. SC and KD conducted the statistical analysis. BN, GM, IP, AP, YB, PR, CS, NS, PB, GM, BP, SD, DP, AR, CT and AB provided data. All authors read and approved the final manuscript.

## Supplementary Material

Additional file 1: Table S1 Predictive factors of remission for 30 patients treated with clofarabine for relapsed or refractory ALL. Click here for file
