# Supplementary material for: French “real life” experience of clofarabine in children with refractory or relapsed acute lymphoblastic leukaemia
Source: Exp Hematol Oncol. 2012 Dec 10;1:39. doi: 10.1186/2162-3619-1-39 (PMC3599405; doi:10.1186/2162-3619-1-39)
Supplement: Additional file 1: Table S1 — Predictive factors of remission for 30 patients treated with clofarabine for relapsed or refractory ALL. [file 2162-3619-1-39-S1.pdf]

Additional file 1: Table S1 Predictive factors of remission for 30 patients treated with clofarabine for relapsed or refractory ALL

Univariable models	OR (95%CI)	P-value
Age (years) at diagnosis	0.99 (0.85-1.17)	0.94
WBC count at diagnosis	0.87 (0.49-1.53)	0.63
Time (years) between diagnosis and relapse	4.07 (1.25-13.3)	0.02

OR (95%CI), odds ratio (95% confidence interval); WBC, white blood cell
